# An amino acid insertion in the PHI loop of the VP2 capsid protein of serotype 1 infectious bursal disease virus deeply modifies its antigenicity and abrogates its propagation into B cells

**DOI:** 10.1186/s13567-026-01749-3

**Published:** 2026-05-20

**Authors:** Cyril Le Nouën, Céline Courtillon, Sébastien Mathieu Soubies, Philip Cruz, Michael A. Dolan, Didier Toquin, Margaux Macaigne, Michel Amelot, Alassane Keita, Hermann Müller, Sonja Härtle, Nicolas Eterradossi

**Affiliations:** 1 French Agency for Food, Environmental and Occupational Health & Safety (ANSES), Ploufragan-Plouzané-Niort Laboratory, VIPAC Research Unit, WOAH Reference Laboratory for Infectious Bursal Disease, Ploufragan, France; 2 RNA Viruses Section, Laboratory of Infectious Diseases, National Institute of Allergy and Infectious Diseases, National Institutes of Health, Bethesda, MD 20892 USA; 3 Bioinformatics and Computational Biosciences Branch., Office of Cyber Infrastructure and Computational Biology, National Institute of Allergy and Infectious Diseases, National Institutes of Health, Bethesda, MD 20892 USA; 4 French Agency for Food, Environmental and Occupational Health & Safety (ANSES), Ploufragan-Plouzané-Niort Laboratory, SELEAC Experimental Department, Ploufragan, France; 5 Faculty of Veterinary Medicine, Institute of Virology, University of Leipzig, Leipzig, Germany; 6 Department of Veterinary Sciences, AG Immunology, LMU Munich, Planegg, Germany; 7 Present Address: INRAE, IHAP, Toulouse University, ENVT, Toulouse, France

**Keywords:** Infectious bursal disease virus, Gumboro, VP2, P_HI_ loop, escape mutant, antigenicity, B cells

## Abstract

Infectious bursal disease of chickens is an economically important disease induced by infectious bursal disease virus (IBDV). Serotype 1 IBDV strains replicate and destroy chicken B cells in the bursa of Fabricius (BF), a primary lymphoid organ essential for chicken immunity. Using a monoclonal antibody selection process applied to a chicken embryo fibroblast (CEF)-adapted serotype 1 IBDV strain, an escape mutant virus that exhibited an extra Glycine residue inserted at position 323 (P_HI_ loop) of its capsid protein VP2 was isolated. This sole amino acid insertion profoundly modified the antigenicity of the virus, which was then unable to replicate in primary chicken bursal cells. Furthermore, infections of chickens with genetically engineered IBDV strains, derived from either a CEF-adapted or a virulent IBDV strain and bearing the same amino acid insertion, did not result in virus replication in the BF, nor histological lesions of the BF, and induced poor seroconversion. Molecular dynamics analyses on VP2 suggested that this insertion reduces the flexibility of the P_HI_ loop and brings this loop in closer contact with the P_BC_ loop. These changes seem critical for IBDV antigenicity and viral infection in bursal B cells but have a limited impact on IBDV propagation in CEF.

## Introduction

Infectious bursal disease (IBD) of chickens (*Gallus gallus*) is an economically important disease induced by the viral agent infectious bursal disease virus (IBDV). Clinical disease is mostly apparent in three- to six-week-old chickens. Various IBDV pathotypes exist, including the so-called very virulent IBDV (vvIBDV) which causes up to 50% mortality in the field [1]. By replicating and lysing its target cell, the immature B cells of the bursa of Fabricius (BF) [2], pathogenic IBDV strains are a major cause of immunosuppression worldwide [3].

IBDV belongs to the genus *Avibirnavirus*, family *Birnaviridae*. It is a non-enveloped double-stranded RNA virus, whose genome consists of two segments designated as segments A and B (3.2 and 2.8 kbp, respectively). Segment A contains the VP5 gene, partially overlapping with a large open reading frame encoding a polyprotein into which the VP2, VP4 and VP3 genes are concatenated. The VP5 protein is involved in virus release and has an anti-apoptotic effect in the early stage of the infection [4]. The VP2 and VP3 proteins are the capsid protein and the ribonucleoprotein, respectively, and constitute the main structural proteins. The protease VP4 induces the proteolysis of the polyprotein and contributes to the maturation of VP2 by progressively trimming its carboxyterminal end. Segment B encodes VP1, the viral RNA-dependent RNA polymerase (RdRp) [3].

The VP2 capsid protein, expressed as a trimer on the virus particle, contains the major antigenic sites responsible for inducing neutralizing antibodies against IBDV. Two serotypes of IBDV, designated as 1 and 2, and several antigenic subtypes within serotype 1 have been defined by cross-neutralization [5] and more recently by antigenic cartography [6]. All pathogenic IBDV strains belong to serotype 1, whereas serotype 2 is apathogenic for chickens [3]. The structure of the 441 aa-long VP2 protein has been determined. VP2 is composed of three domains, namely the base (B), the shell (S) and the projection (P) domains, that all participate in trimer contacts [7]. Mutations significant for IBDV antigenicity are located in the most exposed loops of the P domain, namely at VP2 positions 222 and 223 (in the loop P_BC_, aa 219 to 224), 251 (loop P_DE_, aa 249 to 254), 313 (H □-strand), and 318, 321, 322 and 323 (loop P_HI_, aa 315 to 324) [7–10]. VP2 critical amino acid positions necessary for chicken embryo fibroblasts (CEF) adaptation have been well documented, with the identification of three critical residues in VP2 (aa 253, 279 and 284) [11, 12]. In addition, a motif, conserved among the avibirnaviruses and located in a flexible loop connecting the C and D β-strands at the base of the VP2 P domain (aa 234 to 236, IDA motif), has been identified as a binding motif for the α4β1 integrin [13]. However, other domains responsible for cell receptor recognition and regulating IBDV infectivity in different cell types still need to be identified and localized more precisely to better understand virus-cell interactions.

The present study reports on the unusual insertion of a Glycine residue at position 323 of the P_HI_ loop of VP2 P domain in a replication competent serotype 1 IBDV strain. The selection of a mutated virus, the reintroduction of the mutation by site-directed mutagenesis and reverse genetics, and the characterization of the effects of the mutation on antigenicity, molecular modeling and in vitro and in vivo replication are reported.

## Materials and methods

### Ethics statement

All experiments complied with regulations on animal welfare and animal experiments (authorization no. 22-4 and C-22-745-1 by Préfecture des Côtes d’Armor, France) and followed protocols approved by ethical committee C2EA-16 from the authors’ institution (ComEth Afssa/ENVA/UPEC) under references 10-0007 and 10-0024. Experiments with recombinant IBDVs were performed according to authorization no. 3569 CA-I by the French Commission for Genetically Modified Organisms.

### Viruses

The cell culture-adapted CT strain of IBDV was used in this study. The full-length sequences of both segments have been determined (GenBank accession numbers AJ310185 [14] and AJ310186 [Da Costa, unpublished], respectively) and showed that this strain is closely related phylogenetically to the Cu1 and D78 IBDV strains [15, 16]. All these strains belong to genotype A1bB1 [17]. To produce the virus stock used in the present study, the CT strain was passaged once on a primary CEF culture derived from specific pathogen free (SPF) embryonated hen eggs (Anses, Ploufragan), followed by three passages in the chicken lymphoblastoid LSCC-BK3 cell line (kind gift of Dr Bernard Delmas, INRAE Jouy-en-Josas, France). The resulting supernatant was aliquoted, titrated by serial dilution on CEF and stored at −70 °C until use. The virus titer was calculated according to Reed & Muench formula [18] and was expressed as tissue culture median infectious dose per mL (TCID_50_/mL).

### Antibodies

The production and characterization of the IBDV mAbs have been reported previously [19]. MAbs 1, 3, 4, 5, 6, 7, 8 and 9 all recognize conformation-dependent neutralizing epitopes located in the VP2 protein. Additivity studies have suggested that mAbs 9 and 3, mAbs 3 and 4, mAbs 4, 6 and 7, and mAb 8 probe four different and partially overlapping antigenic domains of VP2. mAb 1 recognizes a fifth antigenic domain that could not be mapped in additivity studies. mAb 20 was used as a non-neutralizing mAb control and recognizes a denaturation-resistant epitope in the VP3 protein [20].

A reference chicken monospecific polyclonal anti-IBDV serum was also used. It was prepared in SPF chickens (ANSES, Ploufragan) inoculated with the Faragher 52/70 IBDV strain (serotype 1) as previously described [21].

### Selection and sequencing of an antibody escape mutant

To select for antibody escape mutants, 75 µL of the CT virus stock were mixed with an equivalent volume of mAb 6. After 2 h of incubation at room temperature, the mixture was added to a suspension of CEF (10^4^ cells/mL). Cells were then incubated at 37 °C with 2% CO_2_ in a 25 cm^2^ culture flask until cytopathogenic effect (CPE) developed. Four days post-infection (dpi), the supernatant was collected, mixed again with mAb 6 as described above, and then submitted to three rounds of cloning by limiting dilution in 96-well CEF culture plates. Finally, the escape mutant to mAb 6 (namely, EM6) was isolated and expanded in CEF to generate a viral stock that was aliquoted, titrated on CEF and kept frozen at −70 °C until use.

The EM6 viral RNA was extracted, reverse transcribed, and its VP2 gene was amplified and sequenced as described previously [16]. The GenBank accession number for the VP2 gene of EM6 is HF544979.

### Molecular modeling and molecular dynamics

The crystal structure of the CT strain VP2 protein was obtained from the Protein Data Bank (PDB ID: 1WCD [7]). Initial models of the protein with the Glycine (Gly) insertion were generated by loop modeling using the program Swiss-PdbViewer 4.1.0 [22].

Molecular dynamics (MD) simulations were performed on both the wild-type model structure and the insertion mutant model under isobaric-isothermal conditions with periodic boundary conditions using the NAMD program (v.2.7) [23]. Each model was explicitly solvated with TIP3P water molecules and Na+ and Cl– neutralizing counterions using the VMD program [24]. Electrostatic interactions were calculated using the Particle-Mesh Ewald summation. The CHARMM27 force field was used with CHARMM atom types and charges [25]. Prior to the start of the simulation, an in silico energy minimization was performed using a conjugate gradient method, followed by slow warming to 310 K in 10 K increments. Each increment ran for 5 picoseconds (psec) in order to equilibrate the system at a given temperature. Production runs were conducted at 310 K for 200 ns (nsec) with data collected every 20 psec. For all simulations, a 2 femtoseconds integration time step was used along with a 12 angstrom (Å) non-bonded term cutoff. Langevin dynamics were used to maintain temperature and a modified Nosé-Hoover Langevin piston was used to control pressure. Distance values from the simulation were obtained every 0.4 ns for 200 ns, which generated 500 measures. The mean values ignored the first 10% of the simulation as the structures were equilibrating during that period. Mean values of Cα-Cα distances are expressed in Å ± standard deviation (SD) value. *This work utilized the computational resources of the NIH HPC Biowulf cluster *[26]*.*

### Construction of the cDNAs with the amino acid insertion G323 into VP2

To investigate the biological effects of the insertion of a Glycine residue at position 323 of VP2, this mutation was introduced by site-directed mutagenesis (Quickchange Site Directed Mutagenesis Kit, Stratagene, primers sequence available upon request) into two different serotype 1 IBDV genetic backgrounds: pACu1 plasmid [27] and pA88 plasmid [28], coding for the segment A of the attenuated Cu1 and of the very virulent 88180 (genotype A3B2) [17], respectively. The sequence of the derived pACu1 ins[G323] and pA88 ins[G323] plasmids were confirmed by Sanger sequencing.

### Rescue of Cu1-derived viruses with and without the VP2 G323 insertion on primary CEF

The pACu1, pACu1 ins[G323] and pBCu1 plasmids were used in reverse genetics experiments to generate rCu1 and rCu1 ins[G323] viral stocks, using a protocol previously reported [28, 29]. Briefly, the plasmids containing the full-length cDNA copies of the native or mutated IBDV genome segments A and of segment B were linearized with restriction enzymes specific for the restriction sites introduced at the 3’ ends of their IBDV inserts. The linearized plasmids were then used as templates for in vitro transcription and capping. The capped cRNAs derived from segments A and B in the combination of interest were transfected into primary CEF. The supernatant of transfected CEF was harvested at three days post-transfection, directly aliquoted and snap frozen before use.

The rescued viruses were then amplified and titrated on CEF. Viral RNA was extracted from an aliquot of each virus as described previously [30]. The coding regions of segments A and B were sequenced using DNA fragments resulting from overlapping RT-PCR (oligonucleotide primers available upon request).

### Growth kinetics of rCu1 and rCu1 ins[G323] on primary CEF and chicken bursal cells

Primary cultures of CEF and primary chicken bursal cells derived from SPF White Leghorn chickens (ANSES, Ploufragan) were prepared on the day before and on the day of the infection, respectively. CEF were seeded in 24-well plates with 5.10^5^ cells per well in Glasgow’s MEM medium (Gibco) supplemented with Tryptose Phosphate Broth (BD), 5% FBS, penicillin (200 IU/mL), streptomycin (0.2 mg/mL) and fungizone (2 μg/mL), and then incubated for 20 h at 37 °C with 1% CO_2_. On the day of infection, the culture medium was removed from the wells, the CEF layers were rinsed once with fresh medium, and infected at a multiplicity of infection (MOI) of 3 with either rCu1 or rCu1 ins[G323] (1 h adsorption at 37 °C with 1% CO_2_). Chicken bursal cells were isolated as previously reported [31] and were directly seeded in 24-well plates and infected with either rCu1 or rCu1 ins[G323] at a MOI of 3, using 5.10^5^ cells per well in Iscove’s modified Dulbecco’s Medium (Gibco) supplemented with all components previously described [31]. Chicken bursal cells were incubated at 40 °C with 5% CO_2_. An aliquot of each inoculum was stored at −70 °C for further titration.

After 1 h of adsorption, the inocula were removed and the cells were washed 3 times with fresh complete medium, before being incubated with fresh complete medium. For chicken bursal cells, which are non-adherent, each washing step included a centrifugation step (at 220 × *g* for 5 min) to recover cells. At 1, 4, 8, 12, 16, 20 and 24 hours post-infection (hpi), the supernatant and the corresponding cells were collected separately, using one well per virus. Supernatants were directly stored at −70 °C for further titration. Viral kinetics assays were repeated three times independently.

Before titration, supernatants were briefly centrifuged (1 min, 12 000 *g*, 4 °C) to pellet cell debris. Supernatants from both CEF and bursal cells were then titrated by serial dilution on CEF as described above. Viral titers were calculated and expressed as TCID_50_/mL [18]. The limit of detection of this method was 10^2.3^ TCID_50_/mL.

At each time point, aliquots of the collected cells were lysed using 350 µL of buffer RA1 (from NucleoSpin RNA kit, Macherey–Nagel) with 3.5 µL of β-mercaptoethanol, and then stored at −70 °C for further genome quantification by real-time RT-PCR [32].

### Propagation of rCu1 and rCu1 ins[G323] in SPF chickens

The propagation of the rCu1 and rCu1 ins[G323] viruses was compared in five-week-old SPF chickens (White Leghorn, ANSES, Ploufragan) in two independent experiments.

In the first experiment (experiment #1), two groups of twelve chickens were housed in negative pressure, filtered-air, BSL2 containment units. Blood samples were collected before inoculation to confirm seronegativity of the chickens against IBDV. Eight birds in each group were then inoculated by the intranasal route with 10^4.6^ TCID_50_ of either virus. The four other chickens in each group were kept uninoculated and acted as contact birds present right from the time of inoculation. All birds were observed daily for clinical signs. At 4 dpi, four inoculated chickens were weighed in each group, euthanized and necropsied. Their BF were weighed and fixed in 10% formaldehyde for subsequent histological analysis. At 22 dpi, the same procedure was repeated with all remaining chickens (four inoculated and four contact chickens per group). Weight measures allowed to calculate the bursa-to-body weight ratio (BBR) to evaluate the BF atrophy level. Histological analysis allowed to quantify the severity of the IBD-induced lesions (bursal lesion score**,** BLS) according to Skeeles’ scale, as previously described [28, 29]. Sera obtained from blood samples in all groups were subjected to virus neutralization (VN) tests.

In the second experiment (experiment #2), two groups of twenty-five chickens per group were housed as described above. Twenty chickens were inoculated with one of the two viruses and five chickens from each group were kept as uninoculated contact birds. At 4 and 10 dpi, five inoculated chickens were studied in each group. Their BF were weighed and then frozen at −70 °C for subsequent quantitative real-time RT-PCR analysis. At 20 dpi in experiment #2, the same samples as already collected at 22 dpi during the course of experiment #1 (see above), were taken on all remaining chickens (ten inoculated and five contact birds per group).

### Quantification of the viral load in samples from animal experiment and growth kinetics studies using real-time RT-PCR

Bursal tissues collected at 4 and 10 dpi in experiment #2 were individually homogenized using Tissue Lyser (Qiagen) under conditions previously specified [33]. RNA extraction was then performed from those bursal homogenates and from lysed CEF and chicken bursal cells collected during the growth kinetics, with the NucleoSpin RNA kit (Macherey–Nagel) following the manufacturer’s instructions. Quantification of IBDV positive segment B strand (B + strand) was performed using a previously described real-time RT-PCR [32] to evaluate levels of viral transcription. The results were standardized either per 50 ng of total RNA for growth kinetics cells, or per gram of BF for bursal homogenates.

### Virus neutralization assays

VN tests were performed either to determine the antigenic profile of the four viruses produced (CT, rCu1, EM6 and rCu1 ins[G323]) using reference mAbs, or to evaluate the specific immune response of IBDV-infected SPF chickens using both the rCu1 and rCu1 ins[G323] viruses as antigens. All VN tests were performed in CEF, using 100 TCID_50_ per well of virus (virus dose confirmed by back titration of the virus stocks), with a previously described protocol [19]. Positive and negative antisera were serially two-fold diluted in the virus suspension. VN titer was expressed as log_2_ of the last serum dilution resulting in 100% neutralization of the CPE as observed at 5 dpi.

### Rescue attempt of 88,180-derived viruses with and without the VP2 G323 insertion in SPF chickens

The pA88, pA88 ins[G323] and pB88 plasmids (designated below as p88 plasmids) were constructed according to previously reported methods [28]. The plasmids were used to attempt the rescue of r88180 and r88180 ins[G323] viruses, respectively. As opposed to Cu1, 88180 does not replicate into CEF. Thus, for each of these two viruses, the supernatants and the scraped transfected CEF were pooled three days post-transfection and were injected intramuscularly to five 7-week-old SPF chickens (same origin and breeding conditions as above). At 4 dpi, the inoculated chickens were euthanized and necropsied. Their BF were sampled for virus isolation on embryonated SPF chicken eggs and for detection of the IBDV genome as previously described [28, 29].

### Statistical analysis

Sets of data were evaluated for significance using the non-parametric Kruskal–Wallis’ test with Dunn’s post hoc test for non-normal data sets. A log_10_ transformation was applied to data sets wherever necessary to obtain equal SD among groups, a requirement for this test. Statistics were performed using the Prism 6 version (GraphPad). Data were only considered significant at *p* ≤ 0.05.

## Results

### The EM6 escape mutant exhibits extensive antigenic changes, due to the sole Gly [323] insertion in its VP2 protein

After three rounds of neutralization with mAb 6, a virus able to replicate on CEF despite the presence of this mAb in the culture medium was isolated and designated escape mutant 6 (EM6). As compared to its parent CT strain, which was efficiently neutralized by the anti-IBDV polyclonal antibody and by all mAbs 1, 3, 5, 6, 7, 8 and 9, the antigenic profile of EM6 was characterized by a lack of neutralization by mAb 6, as expected considering the EM6 selection process (Table 1). However, the absence of neutralization by mAbs 5, 7 and 8 was also noted, as well as a reduced neutralization by mAb 3 and the polyclonal antiserum, thus demonstrating profound antigenic changes in the EM6 virus.

**Table 1:** **Antigenicity of the parent CT strain, the mutant strain EM6 and their reverse genetics-engineered counterparts**

Viruses	G323 insertion	log_2_(Virus neutralization titer) by									
		Polyclonal	Mab 1	Mab 3	Mab 5	Mab 6	Mab 7	Mab 8	Mab 9	Mab 20	Sp2O
CT	No	17	14	19	10	21	23	17	20	< 3	< 3
rCu1	No	16	13	17	9	18	19	14	18	< 3	< 3
EM6	Yes	*13*	14	*11*	** < 5**	** < 5**	** < 5**	** < 5**	17	< 3	< 3
rCu1 ins[G323]	Yes	*14*	16	*12*	** < 5**	** < 5**	** < 5**	** < 5**	17	< 3	< 3

VN titers (the highest dilution of the tested antibody that still neutralizes 100% of the cytopathogenic effect) obtained for the polyclonal antiserum and all monoclonal antibodies are expressed in log_2_. Predilutions of serum and mAbs in VN tests were 1:100 (polyclonal), 1:25 (mAbs 1, 3 and 9), 1:4 (mAbs 5, 6, 7 and 8) or 1:1 (mAb 20), resulting in lower limits of detection being 10, 8, 5 and 3 respectively. The text in bold or italics indicates abolished or reduced neutralization, respectively, as compared with the CT parent virus.

**Figure 1:**
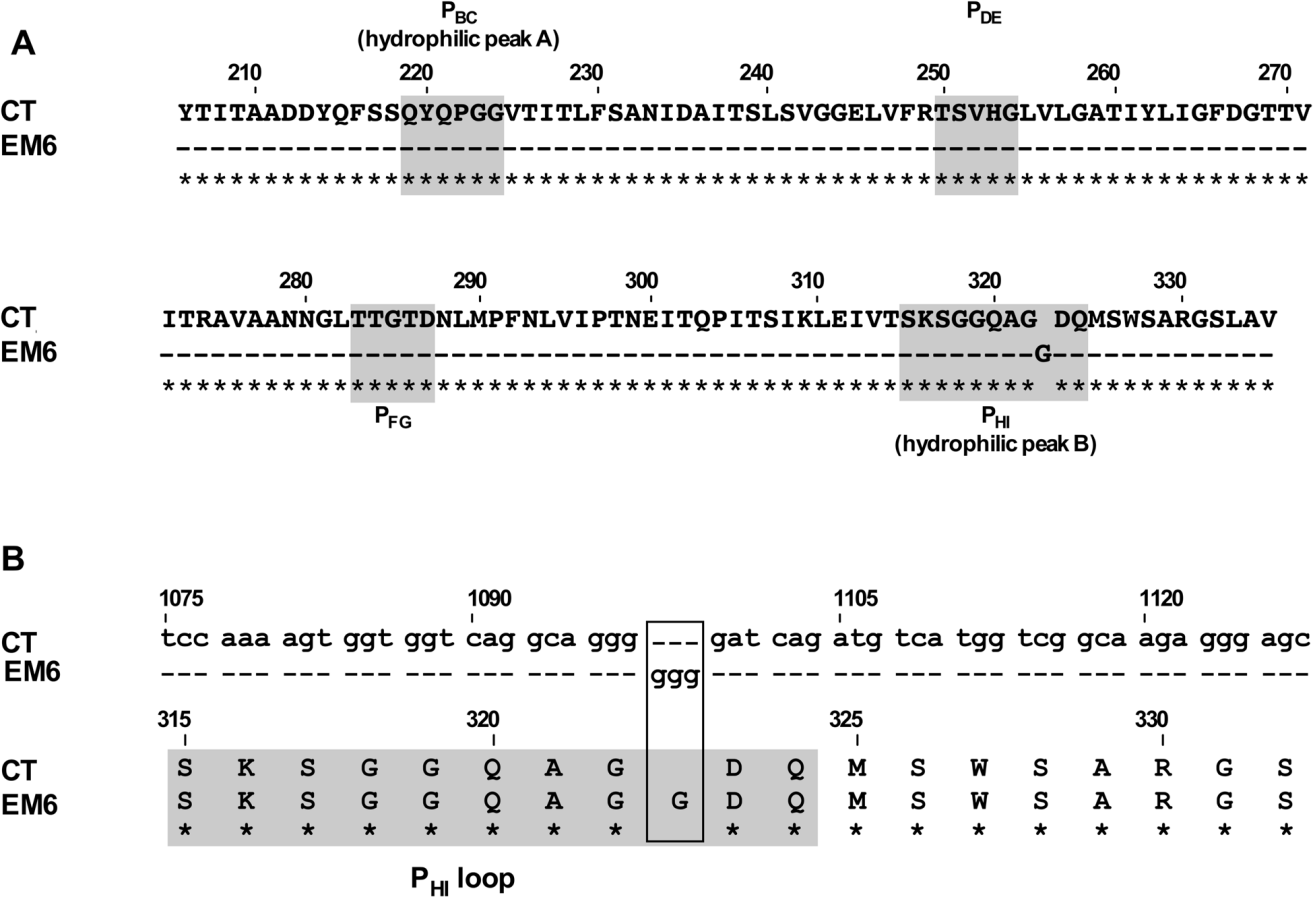
**Partial VP2 amino acid and nucleotide alignments of parent CT and mutant EM6 strains**. **A** Amino acid alignment from VP2 amino acids 206 to 335 including the four loops P_BC_ (hydrophilic peak A), P_DE_, P_FG_ and P_HI_ (hydrophilic peak B). **B** Nucleotide alignment (up) and amino acid translation (down) from nucleotide positions 1075 to 1128 encoding the P_HI_ loop. The « GGG» nucleotides insertion encoding an additional Gly residue in EM6 is boxed. Numbers refer to the amino acid or the nucleotide positions in the segment A or polyprotein of the CT strain (GenBank accession number AJ310185). Dashes indicate amino acid or nucleotide identical to the parent CT strain. Asterisks identify conserved positions. The grey boxes indicate the loops.

The VP2 gene of EM6 was next sequenced. Only the insertion of a Glycine residue at position 323, was identified compared to the parental CT sequence (Figure 1A). This insertion was located in the P_HI_ loop of the P domain of VP2. The aa insertion was encoded by an inserted GGG nucleotide triplet, in frame, in a stretch of four G nucleotides preexisting in the CT parent sequence (Figure 1B).

Using site-directed mutagenesis and reverse genetics, the same mutation was reintroduced in the genetic backbone of another CEF adapted serotype 1 IBDV, namely the Cu1 strain. The parent rCu1 and rCu1 insG[323] (its derivative with the Gly[323] insertion) were rescued by reverse genetics. The sequences of both viruses were checked and did not reveal any nucleotide change, except the intended Gly[323] insertion in the latter virus. In VN assays, the rCu1 and rCu1 insG[323] viruses exhibited exactly the same antigenic profiles as already described in the CT and EM6 viruses, respectively (Table 1), demonstrating that the Gly[323] insertion alone was sufficient to induce the extensive antigenic changes observed in EM6.

### The Gly[323] insertion is predicted to cause structural changes in VP2 projected domain

**Figure 2:**
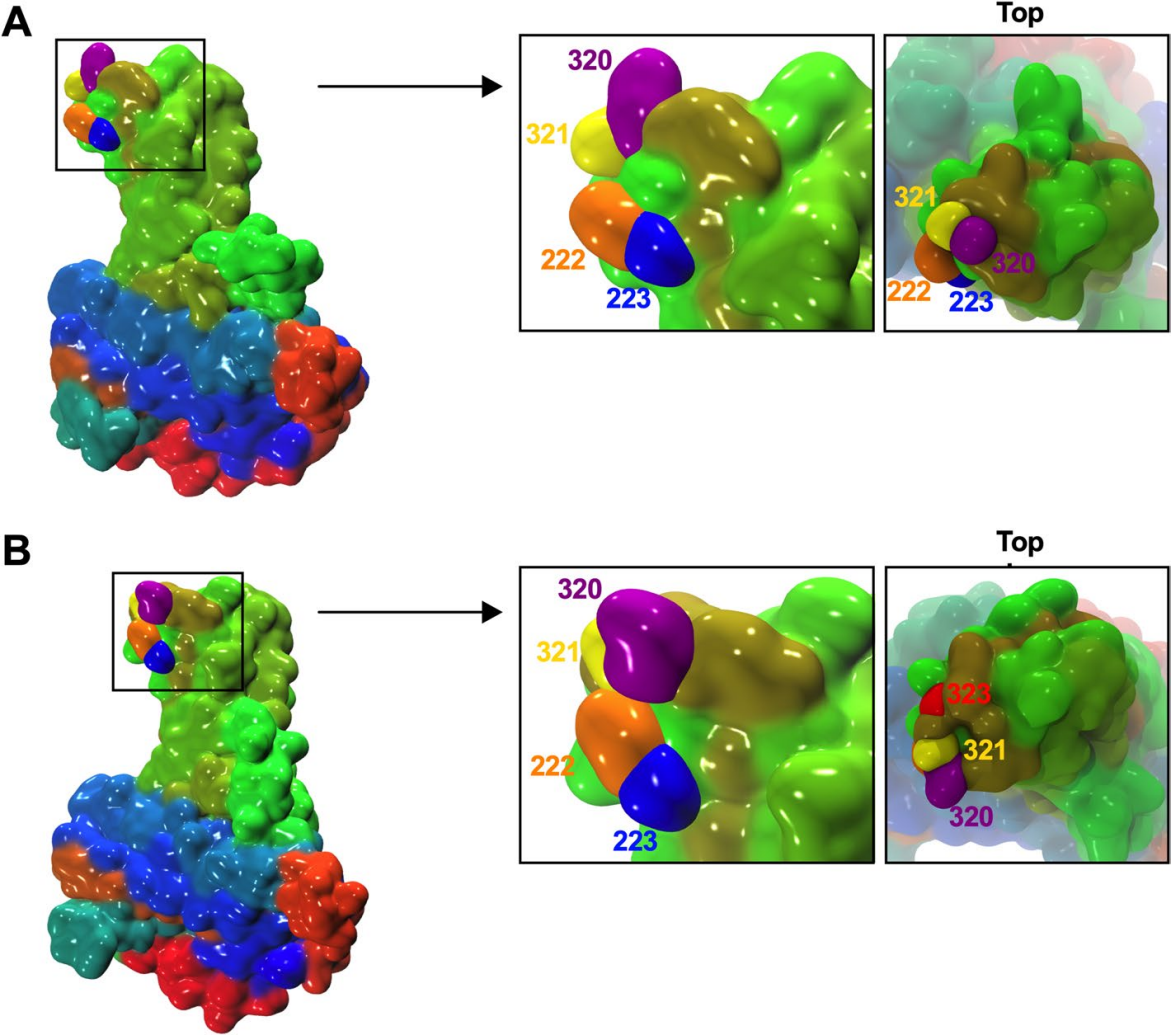
**Structure of a VP2 monomer in the absence or presence of a Glycine insertion at VP2 position 323**. **A** Structure of a wild type VP2 monomer from a typical frame in the MD simulation with a closer view of the region of interest and a top view (boxed). **B** Structure of a VP2 monomer with the insertion of a Glycine at position 323 from a typical frame in the MD simulation with a closer view of the region of interest and a top view (boxed). Amino acids of interest are shown individually: 222 in orange, 223 in blue, 320 in purple, 321 in yellow and 323 insertion in red.

Using molecular dynamics analyses, the potential structural changes induced by the insertion of the Glycine residue at position 323 on the VP2 monomer structure were evaluated in comparison with the VP2 wild-type surface (Figure 2). The insertion was predicted to allow for a loop rearrangement bringing aa 320, 321 and 322 of the P_HI_ loop into closer contact with aa 222 and 223 of the P_BC_ loop, changing the geometry of this region.

This observation was further substantiated by Cα–Cα distance calculations between Q320, A321 or G322 and P222 or G223, which all predicted, in the VP2 protein with the insertion, a statistically significant reduction of distance (*p* < 0.0001) between these aa located in the P_HI_ and P_BC_ loops (Table 2). The closer contact between these loops was predicted to stabilize the entire stem of the structure, making it more rigid throughout the simulation.

**Table 2:** Mean C${\rm{\alpha}}$–C${\rm{\alpha}}$ distances between the indicated amino acids in the CT strain VP2 protein or the mutant VP2 as measured during the molecular dynamic simulations

aa P_HI_ loop	Q320		A321		G322	
aa P_BC_ loop	P222	G223	P222	G223	P222	G223
VP2 wt	8.19 ± 0.78	11.2 ± 0.86	7.53 ± 1.33	10.9 ± 1.27	9.86 ± 0.76	12.8 ± 0.79
VP2-ins[G323]	6.08 ± 0.87	8.43 ± 1.11	5.82 ± 0.79	9.29 ± 0.85	7.04 ± 0.81	10.4 ± 0.71
Difference	2.11^****^	2.77^****^	1.71^****^	1.61^****^	2.82^****^	2.4^****^

C${\rm{\alpha}}$-C${\rm{\alpha}}$ distances are expressed in Å ± SD.

^****^p < 0.0001 using non-parametric Kruskall-Wallis test with Dunn’s post hoc test.

### The Gly[323] insertion abrogates the replication of Cu-1 derived recombinants in chicken bursal cells but not in CEF

**Figure 3:**
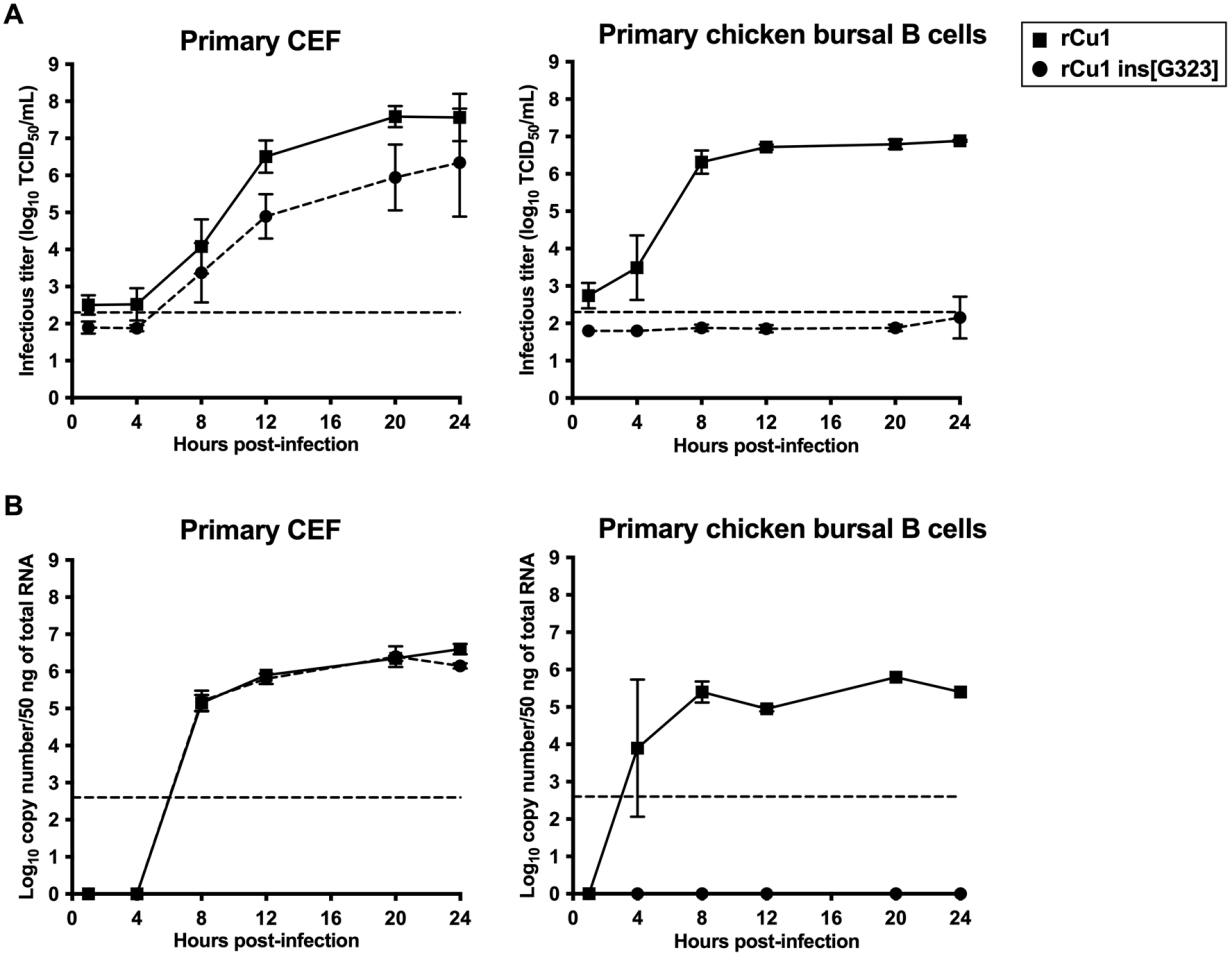
**Growth kinetics of rCu1 and rCu1 ins[G323] viruses on primary CEF and chicken bursal cells**. Replication kinetics (MOI = 3) of rCu1 and rCu1 ins[G323] on primary CEF (left panels) or primary chicken bursal B cells (right panels). Values corresponds to means, and error bars were associated (SD values). **A** Virus was titrated from cell-free supernatants harvested at the indicated time points. The limit of detection (dotted line) corresponds to 2.3 log_10_ TCID_50_/mL. **B** Quantification of B + strand by real-time RT-PCR from cells harvested at the indicated time points. The limit of detection (dotted line) corresponds to 2.6 log_10_ copies per 50 ng of total RNA.

The rCu1 and rCu1 ins[G323] viruses were used for single-step growth kinetics studies performed in CEF and bursal cells. For both viruses and for both cell types, washing steps were effective to the point of making the inocula undetectable or almost undetectable by virus titration at 1 hpi (Figure 3A). On CEF, both viruses replicated efficiently, although slightly less for rCu1 ins[G323], reaching 10^7.57^ and 10^6.35^ TCID_50_/mL, respectively, at 24 hpi. However, only rCu1 replicated in bursal cells, reaching a plateau phase from 8 hpi to the end of the experiment (10^6.31^ and 10^6.88^ TCID_50_/mL at 8 and 24 hpi, respectively), while rCu1 ins[G323] remained undetectable throughout the growth kinetics.

In parallel, the cells collected at the different time points were used to quantify transcriptionally active viruses (Figure 3B). Consistent with the virus titration results, both viruses were able to produce around 10^6^ copies of the IBDV B + strand /50 ng of total RNA on CEF, from 12 hpi onwards. However, on bursal cells, only rCu1 was transcriptionally active (as shown by an average of 10^5.4^ copies/50 ng of total RNA from 8 hpi to the end of the experiment). The virus rCu1 ins[G323] did not produce detectable RNA copies throughout the growth kinetics.

### The Gly[323] insertion prevents bursal replication and virus transmission to contact chickens

**Figure 4:**
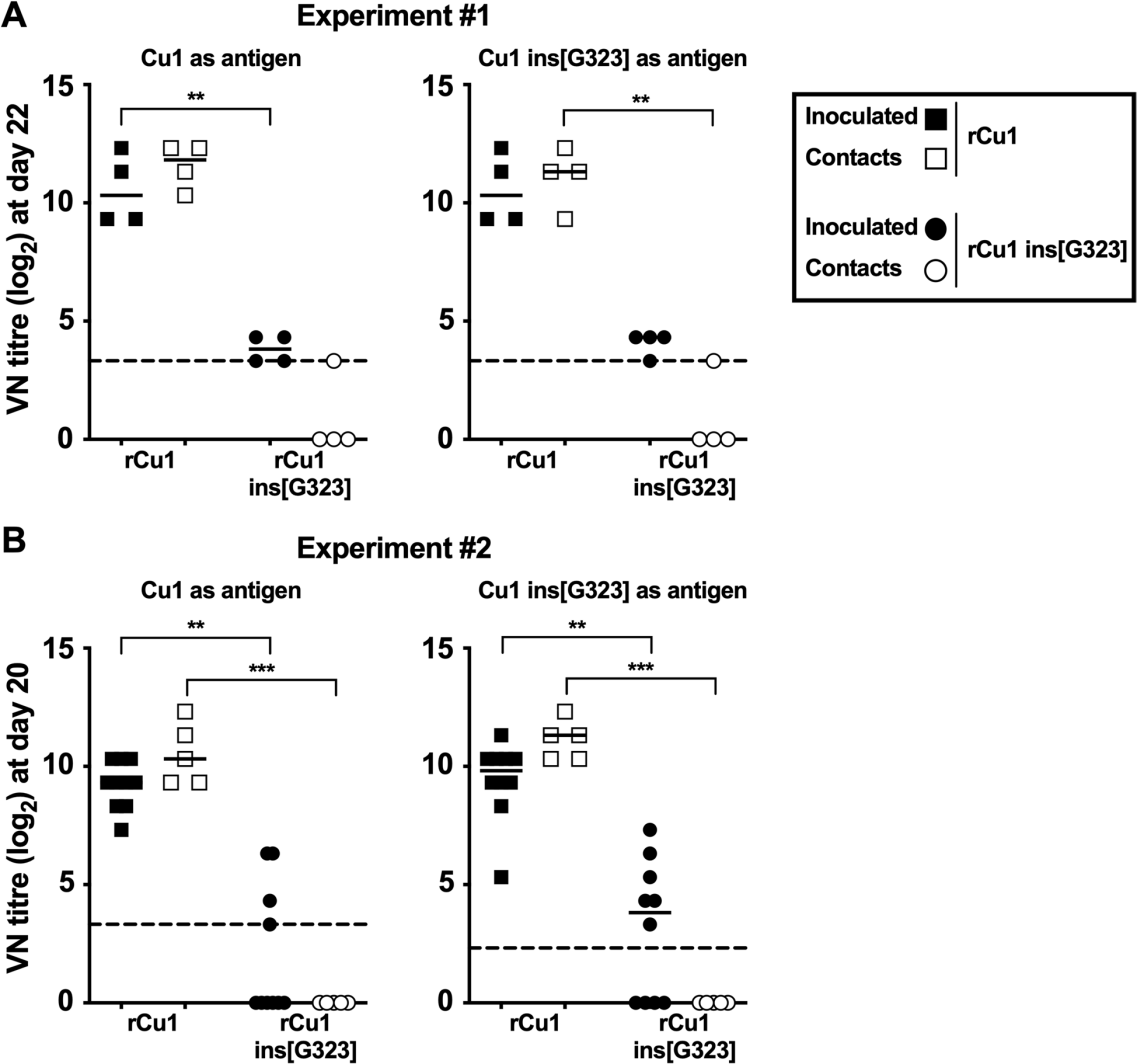
**VN titers of serum collected in rCu1 and rCu1 ins[G323] chicken groups in experiments #1 and #2.** rCu1 (left panels) and rCu1 ins[G323] (right panels) were both used as antigen for VN tests on sera collected from experiment #1 at 22 dpi (**A**) and from experiment #2 at 20 dpi (**B**). Data are expressed in log_2_. The limit of detection (dotted line) corresponds to 3.32 log_2_. Each symbol represents a chicken. The median value is indicated by a bar. Sets of data were evaluated for significance using the non-parametric Kruskal–Wallis test with Dunn’s post hoc test (**p ≤ 0.01, ***p ≤ 0.001).

Neither rCu1 nor rCu1 ins[G323] did induce mortality or clinical signs in chickens in experiments #1 or #2. Infection of the inoculated and contact chickens was therefore evaluated based on their serological responses. VN titers measured in sera collected at 22 and 20 dpi in experiments #1 and #2 respectively were very comparable (Figures 4A and B, respectively). Titers in the rCu1 groups (both in the inoculated and contact chickens) were on average of 11.3 log_2_, thus demonstrating seroconversion in chickens receiving rCu1, and transmission of the rCu1 virus to the contact chickens (left panels). In contrast, VN titers in the rCu1 ins[G323] groups were close or below the limit of detection (3.3 log_2_) in both the inoculated and contact chickens (*p* < 0.01 or *p* < 0.001). The VN results were very similar when the rCu1 ins[323] virus was used instead of rCu1 as a VN antigen (right panels), thus showing that the low antibody levels measured in the chickens receiving the mutated virus were not due to poor antigenic cross reactivity between antibodies elicited by the mutated virus and the rCu1 VN antigen. These low antibody levels suggested limited infection by, and no transmission of the rCu1 ins[G323] virus in SPF chickens.

**Figure 5:**
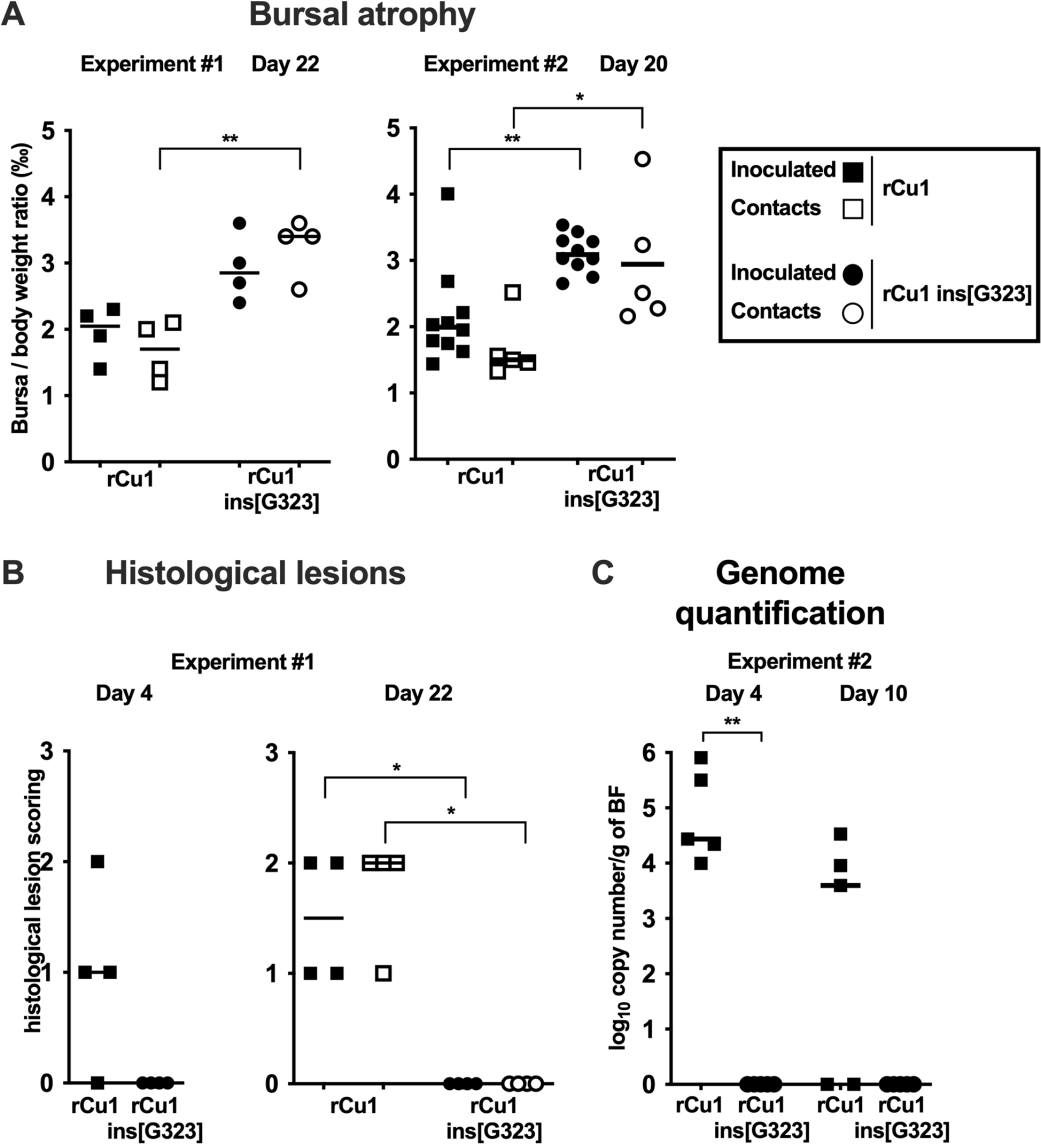
**Bursal atrophy, histological lesions scoring of BF and viral RNA load into BF of rCu1 and rCu1 ins[G323] chicken groups in experiments #1 and #2. A** Bursa to body weight ratio in rCu1 and rCu1 ins[G323] chicken groups (inoculated and contact birds) in experiment #1 (22 dpi, left panel) and #2 (20 dpi, right panel). **B** Histological lesions scoring of experiment #1 BFs collected at 4 dpi (inoculated birds, left panel) and 22 dpi (inoculated and contact birds, right panel) from the rCu1 and rCu1 ins[G323] chicken groups. **C.** Copy numbers of IBDV segment B positive strand in experiment #2 BFs collected at 4 and 10 dpi from Cu1 and rCu1 ins[G323] chicken groups (inoculated birds only). Each symbol represents a chicken. The median value is indicated by a bar. Sets of data were evaluated for significance using the non-parametric Kruskal–Wallis test with Dunns post hoc test (*p ≤ 0.05, **p ≤ 0.01).

Analyses performed on the target organ of serotype 1 IBDV, the BF, were consistent with the conclusion of the serological study. Indeed, the levels of BF atrophy, as measured by BBR in experiments #1 and #2, were very similar in the two experiments (Figure 5A) and significantly more severe in the rCu1 groups (in both the inoculated and contact chickens), than observed in the rCu1 ins[G323] groups (*p* ≤ 0.05 or *p* ≤ 0.01). Consistently with this more severe bursal atrophy, bursal histological lesions (measured in experiment #1 only, Figure 5B) were detected at 4 dpi in 3 out of 4 rCu1-infected chickens, and then in all chickens (both inoculated and contacts) in this group at 22 dpi. In contrast, no histological lesion was observed in the rCu1 ins[G323]-exposed group (neither in inoculated nor in contact chickens) at 4 or 22 dpi (difference in BLS statistically significant at 22 dpi, *p* ≤ 0.05). Finally, the quantification of the IBDV genome in the BF samples collected from inoculated birds at 4 and 10 dpi (experiment #2 only, Figure 5C), was fully consistent with bursal lesions. It demonstrated that viral transcription occurred at both collection dates in the BF of chickens inoculated with rCu1 (median titer of 10^4.4^ and 10^3.6^ log_10_ copies /g of BF, respectively), but not in the bursae of chickens inoculated with rCu1 ins[G323] (*p* < 0.01 at 4 dpi).

Altogether, these results suggested that rCu1 infected SPF chickens, replicated efficiently, induced histological lesions and atrophy of the bursa, and transmitted efficiently to contact SPF birds. In contrast, except a poor seroconversion of infected birds, no sign of infection, bursal replication or transmission to contact birds was observed with the rCu1 ins[G323] mutated virus.

### The Gly[323] insertion also prevents rescue of another serotype 1 non-CEF-adapted IBDV strain in the chicken bursa

To check that the lack of bursal replication observed after the Gly 323 insertion was not strain-specific to Cu1, this insertion was introduced by site-directed mutagenesis into the 88 180 genetic backbone, and the rescue of r88180 and r88180 ins[G323] was attempted into SPF chickens. The in vitro steps of the rescue procedure (plasmid linearization, in vitro transcription and transfection of cRNA) were validated by the relevant controls (presence of a specific band at the expected size for all p88 plasmids, fluorescence of the GFP expression control at 4 and 20 h post-transfection in the transfected CEF) (data not shown). Four days after the intramuscular injection to SPF chickens of the material collected from the transfected CEF, macroscopical lesions were observed on the BF collected for the rescue of r88180, and the r88180 virus was isolated on embryonated SPF chicken eggs with bursal homogenates. In contrast, no bursal lesions were observed in the chickens inoculated for the rescue of r88180 ins[G323], and this virus could not be detected, neither by RT-PCR nor by serial propagation in embryonated eggs, from these bursal homogenates.

These results therefore suggested that the Gly[323] insertion prevented the bursal replication in the genetic context of r88180, as it did with rCu1.

## Discussion

To the best of the authors’ knowledge, this report is the first to describe the spontaneous insertion of an amino acid residue in the VP2 P_HI_ loop of an infectious serotype 1 IBDV strain. In the present case, a Glycine residue was inserted at position 323. A search in publicly available databases, based on 4587 IBDV VP2 nucleotide sequences, revealed a very limited number of sequences with reliable codon insertions in their VP2 gene nucleotide sequences and which are mentioned in peer-reviewed scientific literature. The Miss isolate (accession number AF091098), isolated from a field case in the USA [34], was reported to have a phenylalanine residue (F) inserted between VP2 amino acid positions 164–165. Interestingly, the Miss isolate also presented a modified antigenic profile, as defined with another mAb panel than used in the present study. However, the extent to which the inserted aa might contribute to this feature remains unclear, as VP2 aa positions 164–165 belong to the shell (S) domain of VP2, whereas all aa changes known to date to influence antigenicity are located in the most exposed loops of VP2 projection (P) domain [7]. Noteworthily, the Miss isolate obviously retains its replication ability in the chicken bursa: indeed, this virus was isolated from the bursae of naturally infected chickens and further propagated in vivo in the chicken bursa [34]. Apart from this isolate, the only other IBDV strains known to-date to have a naturally occurring aa insertion in VP2 are the serotype 2 strains. They all exhibit either an additional Serine (S) residue (e.g., 23/82 or OH strains, accession numbers AJ878913 or U30818, respectively) or an additional Asparagine (N) residue (e.g., JG031/KEN/15 or H286/17/Poland/2017 strains, accession number KY450713 or OP978056, respectively) at VP2 aa position 248, which precedes the P_DE_ loop motif.

The four loops P_BC_ (aa 219 to 224), P_DE_ (aa 249 to 254), P_FG_ (aa 283–287) and P_HI_ (aa 316 to 324) are all located in the outermost part of the VP2 protein [7, 8], making them the most exposed regions where antigenically significant aa changes between IBDV strains do cluster. The flexibility to accommodate aa variations in the four VP2 loops led to the hypothesis that the loops might be flexible enough to also accept aa insertions. This hypothesis was addressed experimentally by using IBDV reverse genetics [12] or VP2 expression plasmids to introduce and possibly express foreign epitopes in recombinant VP2 proteins. Such an approach would allow the use of IBDV particles or virus-like particles as antigen carriers. The P_BC_ loop was shown to tolerate a 12-aa insertion and retain immunogenicity as a recombinant VP2 protein [35, 36]. A recombinant IBDV with a 9 aa-long insertion (NDV epitope) between amino acid positions 221 and 222 in the P_BC_ loop was also successfully rescued [37]. In contrast, insertions of foreign epitopes in the P_HI_ loop prevented efficient expression and assembly of the recombinant VP2 protein [35] or impaired the rescue of the resulting recombinant IBDVs [37, 38]. In the present paper, a replication-competent IBDV strain with an extra Glycine residue in its P_HI_ loop was isolated. This extra glycine has been reintroduced by site-directed mutagenesis and reverse genetics into the Cu1 strain. It will be interesting to investigate if the modified P_HI_ loop of the mutant virus offers more opportunities for the insertion of additional epitopes. This might be the case between the two consecutive Gly residues now observed at VP2 positions 322 and 323 in EM6 and rCu1 ins[G323].

The molecular mechanism that led to the insertion observed in EM6 and reproduced in rCu1 ins[G323] remains to be established. Sequence comparisons between the parent and mutant viruses revealed that the extra Glycine residue was encoded by a “GGG” nucleotide triplet inserted in frame, in a stretch of four G nucleotides preexisting in the sequence of the parent virus. Such a situation is reminiscent of the mechanisms described during the transcription of the Paramyxovirus phosphoprotein messenger RNA (mRNA), where insertions of one or more Gs by the RdRp were shown to always occur within a short run of Gs [39]. A stuttering mechanism has been suggested in Paramyxoviruses, by which the viral polymerase iteratively copies a template C residue during mRNA synthesis [39]. Whether or not polymerase stuttering also occurs in IBDV awaits further study.

The Gly[323] insertion event observed in the P_HI_ loop of VP2 in the context of two replication competent IBDVs, the EM6 and rCu1 ins[G323] viruses, provided a unique opportunity to investigate the consequences of this insertion on VP2 structure, on IBDV antigenicity and on the virus replication competency. VP2 position 323 has been documented as being a key position able to influence the structure of VP2. Previous modeling analyses suggested that an aspartic acid to glutamic acid mutation at this position (D323E) might alter the structure of the region and its associated electrostatic potential, and might break a stabilizing salt bridge with a lysine at position 316 [8, 40]. The modeling analyses performed in the present study suggested another possible impact on VP2 structural changes occurring at position 323, as the glycine insertion was predicted to reduce the flexibility of the P_HI_ loop, bringing it in closer contact with the P_BC_ loop.

Regarding antigenicity, the Gly[323] insertion abolished virus neutralization by mAbs 5, 6, 7 and 8, and reduced virus neutralization by mAb 3, thus affecting at least two non-overlapping antigenic domains, previously defined in additivity studies [19]. The binding of mAbs 6 and 8 had already been linked with VP2 critical amino acid positions 318 to 324 [30], and appears quite consistently to be also affected by the Gly[323] insertion. More surprisingly, neutralization by mAb 3, whose binding had previously been shown to critically depend on amino acids 222–223 in the P_BC_ loop [30], was also reduced in EM6 and rCu1 ins[G323]. Since the G323 insertion was predicted to bring P_HI_ closer to P_BC_, it could be hypothesized that these changes results in steric hindrance and reduced access to the P_BC_ loop in the EM6 and rCu1 ins[G323] viruses, thus explaining these viruses reduced neutralization by mAb 3. These overall findings are consistent with other studies demonstrating that VP2 position 323 has a profound influence on IBDV antigenicity. Indeed, changes at this position altered binding of different monoclonal antibodies such as Mab 10, Mab 57 [8, 10, 41] and Mab 2-5C-6F [40], or reduced the neutralizing activity of a polyclonal antiserum [8, 40], a trend also present in our VN results (Table 2).

Finally, in addition to significant structural and antigenic consequences, the Gly[323] insertion also had a striking impact on IBDV replicative capacities in host cells. The insertion-bearing EM6 and rCu1 ins[G323] viruses retained the ability to replicate in CEF, as demonstrated by their selection or rescue process, respectively, and by the growth curve study for the latter. However, although rCu1 ins[G323] was still able to replicate in CEF, its maximum titer was 17-fold lower than that of the parental rCu1 virus. These results are in line with previous studies showing that amino acid changes in the P_HI_ loop at positions 318, 321 and 323 negatively influenced IBDV replication on chicken embryo cells [8]. These observations support the hypothesis of Letzel et al. who proposed that changes in the P_HI_ loop may influence the ability of VP2 to bind to IBDV receptors, as it is adjacent to the P_DE_ loop which is involved in particle–particle interaction and modulates the adaptation of the virus to CEF culture [42]. Interestingly, the only replication-competent recombinant IBDV rescued after the VP2 insertion of a foreign epitope (between position 221 and 222 in the P_BC_ loop) also exhibited a 0.5 to 1 log_10_ reduction of its maximum virus titer, as compared to its parent virus. The engineered virus retained however the ability to replicate in the chicken bursa [37].

In our study, while the mutated virus was still able to replicate in CEF, the Gly[323] insertion prevented the infection of primary chicken bursal cells, as well as replication in the BF of chickens in in vivo experiments. No RNA was detected in either case, no viral particle was titrated in the growth kinetics studies, and no histological lesion was scored on BF from infected chickens. This cell tropism abrogation was not strain-specific as bursal replication of the vvIBDV-related 88,180 strain was also impaired when the G323 insertion was engineered into the genetic backbone of this non CEF-adapted IBDV strain. It is worth mentioning here that the only other IBDV strains identified so far which are not able to replicate in bursal cells are the serotype 2 IBDV strains [27, 31]. Remarkably, serotype 2 strains also exhibit an aa insertion in VP2.

Although progress has been made in understanding cellular components involved in the IBDV receptor, its exact nature is still unclear. Chicken B lymphocytes are IBDV natural target cells. N-glycosylated proteins [43], the λ light chain of sIgM-bearing B lymphocytes [44], the chicken transmembrane protein CD74 [45] or the chicken CD44 isoform [46] have all been suggested to play a role. vvIBDV do not infect 293 T or DF1 cells. However, the recombinant expression of chicken CD44 on the surface of 293 T or DF1 cells, originally lacking the expression of this protein, allows entry of vvIBDV [46]. In chicken fibroblasts, to which IBDV strains can be adapted provided their VP2 exhibits critical amino acids at positions 253, 279 and 284 [11, 12, 47, 48], four other molecules have been reported to be involved in IBDV binding: chicken annexin II [49], chicken heat shock protein 90 (cHSP90) [50], chicken heat shock cognate protein cHSC70 [51], as well as the α4β1 integrin heterodimer [13], a highly abundant integrin also found in immature lymphocytes [52]. An earlier study suggested that the interaction of the different IBDV serotypes with CEF and B lymphocytes might involve three different receptors, possibly co-expressed [53]. Identifying which chicken proteins interact with IBDV VP2, with or without the Gly[323] insertion, may help clarify which cellular factors are critical for IBDV permissivity in chicken B lymphocytes.

In conclusion, a spontaneous Glycine insertion at aa position 323 in the P_HI_ loop of the capsid protein VP2 in a serotype 1 IBDV strain induced VP2 structure changes, profound antigenic modifications, altered only slightly virus replication in CEF but abrogated the infection of B lymphocytes, the normal cell target of serotype 1 IBDV. It is not clear whether a similar mutation could occur under natural circumstances. Indeed, the immune pressure built by post-infectious or post-vaccinal antibody responses targets a broader set of epitopes than the mAb 6 selective pressure used here to select EM6. In addition, reduced replication kinetics as observed in rCu1 ins[G323], and the loss of the bursal target, suggest that, should such mutant viruses occur *in natura*, they might be quickly outgrown by their parent serotype 1 viruses which have a better fitness. However, although obtained under a laboratory environment, these mutant viruses and the related findings help documenting which VP2 motifs are critical for serotype 1 IBDV antigenicity and interactions with its target cells.

## Data Availability

The datasets used and/or analysed during the current study are available from the corresponding author on reasonable request.
